# Fear of Missing Out, Mental Wellbeing, and Social Connectedness: A Seven-Day Social Media Abstinence Trial

**DOI:** 10.3390/ijerph17124566

**Published:** 2020-06-24

**Authors:** Lorna Brown, Daria J. Kuss

**Affiliations:** International Gaming Research Unit and Cyberpsychology Research Group, Psychology Department, Nottingham Trent University, 50 Shakespeare Street, Nottingham NG1 4FQ, UK; lrbrown1218@gmail.com

**Keywords:** social media use, social media abstinence, smartphone use, fear of missing out, mental wellbeing, social connectedness, social media addiction, social networking

## Abstract

Smartphones aid the constant accessibility of social media (SM) applications, and these devices and platforms have become a key part of our everyday lives and needs. Previous research has focused on the psychological impact of social media use (SMU) and SM abstinence has only received limited attention. Therefore, employing a combination of an experimental within-subjects mixed methodology using surveys to obtain both quantitative and qualitative data, this study aimed to compare psychosocial factors of fear of missing out (FoMO), mental wellbeing (MWB), and social connectedness (SC) before and after seven days of SM abstinence. Results revealed that participants (*N* = 61) experienced a significant increase in MWB and SC, and a significant decrease in FoMO and smartphone use following SM abstinence. There was a significant positive relationship between MWB and SC change scores and a significant negative relationship between SC and FoMO change scores. There were no significant differences in levels of SMU before abstinence or across genders in FoMO, MWB, and SC change scores. Thematic analysis revealed coping, habit, and boredom as motivations for SMU, and notification distractions presenting a challenge for successful abstinence from SM. Participants indicated that abstinence resulted in the perceived need to fill their time with non-SM applications. Finally, thematic analysis revealed mixed experiences of perceived connectivity in the absence of SMU. Findings present implications for the importance of unplugging from SM for temporary periods because scrolling through SM to fill time is a key motivator of SMU, and notifications encourage SMU and trigger FoMO.

## 1. Introduction

The present day and age is described as the ‘smartphone era’, with smartphones ubiquitously integrated into our everyday lives [1,2]. These digital devices are now used in place of desktop computers to perform everyday tasks, such as sending and receiving emails [3], providing permanent accessibility to any application and opportunity to communicate at our fingertips [4]. Smartphones also provide access to health applications to encourage users to participate in various health and wellbeing programmes [5]. However, recent statistics report that the most used smartphone applications are social media (SM) [6], and their use has dramatically increased over the last decade [7]. Social media is defined as the “interactive web and mobile platforms through which individuals and communities share, co-create, or exchange information, ideas, photos, or videos within a virtual network” [8] (p. 2). Research highlights a difference between SM and messaging-only applications, such as WhatsApp, which function as text messaging tools and thus constitute a separate domain from SM [7,9,10].

Described as the ‘extension of man’ [11], SM has become almost inescapable, and it has now become the norm for individuals to own accounts on multiple SM platforms [12]. Although constant access to these platforms has made communication more efficient, health-related concerns of social media use (SMU) are at the forefront of research on technology use due to the wide availability and use of smartphone SM applications and constant receipt of regular notifications and updates [13,14].

Defined as a “pervasive apprehension that others might be having rewarding experiences from which one is absent” [15] (p. 1), fear of missing out (FoMO) is classified by the desire to be constantly connected with what other people are doing. FoMO is a strong motivator for SMU [16], and may impact wellbeing. The concept of wellbeing, how people feel and function [17], is of interest to researchers in the domain of technology use [18]. Positive mental wellbeing (MWB) is the state in which “the individual realises his or her own abilities, can cope with the normal stresses of life, can work productively and fruitfully, and is able to make a contribution to his or her community” [19] (p. 49). Furthermore, one’s sense of social connectedness (SC) is highly valued because of the importance of authentic connections [20]. SC is described as an individual’s emotional connectedness with themselves, others, and society, with the need to maintain a sense of belongingness and positive social relationships [21,22]. The continued search for causal drivers of FoMO, MWB, and SC seems paramount since they impact an health, life satisfaction, and wider society [23,24].

Most previous research has focused on the associations between SMU and perceived FoMO, MWB, and SC, rather than cause and effect [25]. Although some researchers claim individuals who score higher in perceived FoMO are driven to use SM, these studies are usually characterised by samples with high anxiety who depend on SM to compensate for lack of offline relationships and to maintain a sense of belongingness [12,26].

Przybylski et al. [15] and Oberst et al. [27] indicate that SMU and FoMO have a reciprocal relationship, where perceived FoMO drives SMU to fulfil psychological needs of belongingness, reinforcing SMU. This is supported by self-determination and uses and gratifications theory, where reinforcements and rewards, in the form of SM notifications and validation through ‘likes’, explain the continual and habitual nature of SMU [28]. In a culture constantly attached to smartphones, these SM rewards and reinforcements are limitless in both access and receipt, and users are constantly subject to new information and updates. Researchers state that SMU exacerbates an individual’s perceived FoMO [4,29], suggesting stronger evidence for a cause and effect relationship whereby SMU increases FoMO. Moreover, the perceived obligation to maintain connections and stay updated on SM has been described as “technostress”, and can negatively affect one’s wellbeing [30,31].

Hunt et al. [32] found causal evidence that limiting SMU to ten minutes per platform per day for three weeks significantly decreased loneliness and depressive symptoms, FoMo and anxiety. However, their experiment included a control group where some subjects abstained and others did not, making it difficult to compare use and abstinence in the same individual. Primack et al. [33] found that participants with higher SMU felt more socially isolated than participants with lower SMU, demonstrating a positive relationship between SM engagement and feelings of isolation. Further, research shows that face-to-face interactions act as a buffer to prevent negative MWB in comparison to online interactions [34], suggesting that offline relationships promote a more positive sense of wellbeing than online. However, Grieve et al. [35] argue that the benefits of face-to-face SC can translate online, with Facebook SC positively relating to perceived MWB, although this research makes no comparisons between SMU and SM non-use; thus, a clear causal relationship cannot be determined [36]. Longitudinal research comparing SM use with SM non-use found that SM users are more likely to report higher levels of loneliness than SM non-users, thereby negatively affecting perceived MWB and SC [37].

SMU is appealing due to the sense of being connected that it provides [30,35]. Scholars question whether this sense of socialisation is an illusion, with face-to-face interactions creating more meaning and providing authenticity compared with online interactions. A study exploring why regular Twitter users take breaks from the site found that most individuals’ motivations for abstinence periods are due to concerns over the disconnect between online and offline [38], demonstrating that individuals perceive offline interactions as more important than online interactions.

With SMU now entwined with our daily routines via smartphones [1,2], SM abstinence allows exploring the tangible effects of SM on people’s lives [39] as little is known about SM abstinence and the potential challenges associated with it [38]. SM abstinence may increase participants’ MWB and SC, and decrease FoMO, where higher SM users who abstain will notice these changes more than lower SM users.

There have been very few studies on SM abstinence and fewer employing an experimental design to determine cause and effect [24,36]. Roberts and Koliska’s [39] study investigated students going media-free for 24 h and found distress was the most common experience, including high FoMO and feeling isolated. These negative responses may be due to abstaining from all media including messaging, considered an essential communication tool vital for many young people’s sense of safety [9,10,40]. Furthermore, heightened distress and feelings of isolation may be a result of the exceptionally short abstinence period. Consistent with addiction and dependency literature, anxiety and distress are usually heightened at the beginning of abstinence where initial withdrawal is common with reduced reinforcements, but it often decreases over time with adaption when individuals learn to satisfy their psychological needs elsewhere [12,41]. Research also revealed that some participants failed to fully abstain due to inadvertent use, demonstrating the habitual nature of media use, warranting further exploration.

A seven-day Facebook abstinence study revealed a significant reduction in perceived stress following abstinence in comparison with a non-abstaining control group, where reduced stress was greater in more excessive SM users [42] because the positive effects of abstinence were due to the seven-day period being long enough for positive adaption to the change, and short enough to be feasible for participants to maintain abstinence [43]. Consistent with a study exploring 99 days of Facebook abstinence, participants found their compulsion to check the site was stronger in the initial stages of abstinence and this reduced over time for those who fully complied [44]. Another seven-day Facebook abstinence study provided causal evidence that a Facebook abstinence period increased life satisfaction and elicited more positive emotions in comparison to a control group of participants who continued using the site, and positive effects in the abstinence group were significantly greater for more excessive users [24].

Research on SM abstinence is scarce and the aforementioned studies focused on Facebook use rather than multiple SM platforms [45] and used self-reports to measure SMU [25]. As multiple SM platforms are now primarily accessed through portable smartphones [12] and SMU self-report measures tend to be subjective and biased [46,47,48], multiple SM platform use needs to be studied with more objective measures over time. The aforementioned studies on SM abstinence employed a between-subjects experimental design, but a within-subjects experimental design is more beneficial in reducing errors associated with individual differences [25,49,50,51]. Furthermore, as little is known about regular SM users taking on abstinence periods and the associated challenges, a mixed-methods approach may provide insight into individual abstinence experiences and theoretical implications [38].

This research aims to bridge the gap in SM abstinence research by using a novel combination of mixed methods, employing a within-subjects experiment to compare behavioural effects of a seven-day SM abstinence period across multiple SM platforms, measuring SMU with smartphone applications objectively. The following hypotheses were formulated:

**Hypothesis 1 (H1).**  *Participants’ baseline SMU will be significantly positively correlated with changes in FoMO, MWB, and SC*.

**Hypothesis 2 (H2).**  *There will be a significant decrease in participants’ perceived FoMO*.

**Hypothesis 3 (H3).**  *There will be a significant increase in participants’ perceived MWB*.

**Hypothesis 4 (H4).**  *There will be a significant increase in participants’ perceived SC*.

**Hypothesis 5 (H5).**  *There will be a significant decrease in participants’ mean hours per day spent on their smartphone*.

Moreover, the qualitative part of this study aims to uncover challenges and experiences of a seven-day abstinence period from SMU.

## 2. Materials and Methods

### 2.1. Participants

G*Power analysis [52] was performed, calculating a required sample of at least 45 participants to test the hypotheses at a significance level of 0.05 and power of 0.8, using a Bonferroni-adjusted alpha level because of multiple testing. An opportunity sample (*N =* 78) was recruited over a two-month period via SM to take part in the seven-day abstinence trial. Criteria required participants to be ≥18 years of age, own a smartphone, regularly use SM (i.e., most days in a given week), have or download a working application to their smartphone as a measure of phone use and SMU (see Section 2.3), and be willing to abstain from SM for seven days. During data cleaning, one participant was removed who did not agree to consent clarifications and a further three were removed who did not meet participant criteria. An additional 13 were removed who did not fully abstain from SM. The final analyses included a total of 61 participants.

Of the final participants (*N* = 61), 67.21% (*N* = 41) were female, and 32.79% (*N* = 20) were male. The age range was 20 to 49 years (*M* = 24.4, *SD* = 4.95). The sample included a wide range of SM users, with a SMU range between 0.5 and 55.5 weekly hours (*M* = 10.5, *SD* = 11.01 weekly hours), measured by participants’ total number of hours spent on SM altogether during seven days of SMU before abstinence. Hours spent on SM before abstinence were considered weekly rather than daily to gain an overall insight into participants’ SMU throughout a seven-day period of “normal” use. In addition, one of the main features of the applications used to measure SMU displays a visual order of most used to least used SMU platforms (see Table 1) to better represent participants’ usage over a seven-day period as opposed to day-to-day usage. Furthermore, the sample was representative of the wider population in the type of SM users included, with Facebook, YouTube, and Instagram being the most used platforms by participants, and smartphones being the most used device to access SM [3,53], as shown in Table 1.

### 2.2. Design and Procedure

This within-subjects design used a mixed-methods approach, gathering both quantitative and qualitative data. After ethical approval from the Research Ethics Committee at a UK University, two online surveys using Qualtrics survey software (Qualtrics, Provo, UT) were designed for participants—the first after seven days of using SM as normal (before abstinence), and the second after the seven-day SM abstinence trial (after abstinence).

#### 2.2.1. Pre-Abstinence

Informed consent was obtained, and research information including guaranteed data anonymity and participant criteria was given. Participants were asked to create a unique identifier which they would remember and use again for the second questionnaire for pre- and-post-abstinence data to be matched for each participant. This survey consisted of questions about participants’ smartphone and SMU, and perceived FoMO, MWB, and SC. Open questions at the end of the survey asked participants about how they felt, and their anticipated challenges about abstaining from SM for seven days.

Upon completion of the pre-abstinence survey, participants were required to contact the researcher and begin the seven-day SM abstinence period upon completion of which they were sent a link to the post-abstinence survey.

#### 2.2.2. Post-Abstinence

At the end of the seventh day, participants were contacted with the link to the post-abstinence survey, which consisted of questions about participants’ smartphone use and SMU, and perceived FoMO, MWB, and SC. Open questions at the end of this survey asked participants how they felt during the seven-day period of SM abstinence and what they found difficult.

#### 2.2.3. Qualitative Data

Thematic analysis, as the most suitable method for written data [54], was performed on participants’ responses (*N* = 61) to the open questions in both surveys to conceptualise their thoughts and feelings, including their anticipated challenges and retrospective experiences of the SM abstinence trial. To explore participants’ anticipated challenges associated with the seven-day SM abstinence trial, open questions in the pre-abstinence survey included “How are you feeling about the fast?” and “How do you feel it will challenge you?” To explore participants’ retrospective experiences of the seven-day SM abstinence trial, open questions in the post-abstinence survey included “Did you manage to abstain? How did you feel? What made this difficult?” In the recruitment process, the study was advertised as including an “abstinence period” as well as a “fast from social media”.

Based on the work of Braun and Clarke [55], participants’ written responses were read over multiple times for familiarisation before semantic codes were captured by looking for consistent patterns in the data. Codes were reviewed by rereading the data to ensure that they were accurate and consistent. The uncovered patterns and their significance were theorised, and broader meanings explored.

As a novel research focus on SM abstinence, an inductive approach allowed for openness and new theory to emerge, and semantic coding was used where no meaning or idea was assumed beyond what participants explicitly stated, involving explicit and surface meaning to uncover common patterns [55].

### 2.3. Measures

Social media was defined based on current statistics which revealed that the most used SM platforms in the UK to produce, share, co-create, and exchange information [8] are (in descending order): Facebook, YouTube, LinkedIn, Instagram, Snapchat, Twitter, Pinterest, Tumblr, and Reddit [53]. However, LinkedIn was not considered SM for this study, as its primary function serves professionalism and the site is considered a necessity for applying for jobs [56], and thus abstaining from LinkedIn use was considered not feasible. Messaging-only applications such as Facebook Messenger and WhatsApp were also not included in the definition of SM as they are considered social networking, distinct from SM [7]. Further, these applications are now treated as vital communication tools, often used in place of traditional text messaging [9,10], and thus would not be practical for abstinence.

#### 2.3.1. Smartphone Social Media Use and Smartphone Use

Smartphone SMU was measured using the Screen Time application (for iPhone users) and the ActionDash or Tracky application (for Android phone users). These applications also measured participants’ mean hours spent on their smartphone per day. Participants input data from these applications into the surveys, avoiding self-report measures of smartphone SMU which tend to be unreliable and subject to cognitive bias [46,48].

Screen Time, the new application included in the iOS 12 update, provides a report detailing mean daily hours spent on smartphones, and time spent on each SM application in the last day and in the last seven days. ActionDash and Tracky are versions of the Digital Wellbeing and Screen Time helper for Android. These applications provide a daily and weekly breakdown of how long users spend on applications per week, and mean daily hours spent on their smartphone.

Participants were asked to provide their average number of daily hours on their smartphone in the last seven days, and the total number of hours spent on each SM application in the last seven days to the nearest quarter of an hour to take into account momentary inadvertent access to SM applications [39]. For example, if the application calculated the total number of hours spent on Instagram in the last seven days as one hour and 40 min, participants would input this as 1.75. If participants did not currently have one of the applications, they were asked to download it and only begin the experiment and respond to the first survey once it had collected seven days’ worth of data.

#### 2.3.2. Other Device Social Media Use

SM accessed on any other digital device (e.g., desktop or tablet) was measured using self-report, where participants were asked to input their estimated total number of hours on each SM site in the last seven days for both surveys. Although this was measured through participant self-report, SM accessed through other digital devices is likely to be more intentional and less than smartphone SMU, and is therefore more accurately remembered [3,47].

#### 2.3.3. Fear of Missing Out

Fear of missing out was measured using the 10-item Fear of Missing Out Scale (FoMOS) [15]. Participants respond to statements measuring the extent to which they fear missing out on various events or experiences. Items are all positively worded and rated on a five-point Likert scale (1 = *Not at all true of me*, 2 = *Slightly true of me*, 3 = *Moderately true of me*, 4 = *Very true of me*, 5 = *Extremely true of me*). A higher FoMO score indicates a higher perceived fear of missing out. The scale forms a reliable measure (Cronbach’s alpha = 0.87 to 0.90) [15].

#### 2.3.4. Mental Wellbeing

Mental wellbeing was measured using the 14-item Warwick–Edinburgh Mental Well-Being Scale (WEMWBS) [57]. Participants respond to statements that measure subjective wellbeing and psychological functioning. Items are all positively worded and rated on a five-point Likert scale (1 = *None of the time*, 2 = *Rarely*, 3 = *Some of the time*, 4 = *Often*, 5 = *All of the time*). A higher WEMWBS score indicates higher perceived mental wellbeing. Studies have confirmed validity, internal consistency, and test-retest reliability in general populations (Cronbach’s alpha = 0.89) [58,59,60].

#### 2.3.5. Social Connectedness

Social connectedness was measured using the 20-item Social Connectedness Scale—Revised version (SCS-R) [21,61]. Participants respond to statements that measure the ways they feel connected to others in their environment on a six-point Likert scale. For this current research, a mid-way scale point of ‘Neither Agree nor Disagree’ was added, making this a seven-point scale (1 = *Strongly Disagree*, 2 = *Disagree*, 3 = *Mildly Disagree*, 4 = *Neither Agree nor Disagree*, 5 = *Mildly Agree*, 6 = *Agree*, and 7 = *Strongly Agree*) to be consistent with the WEMWBS and FoMO scales to consider respondents who may hold a neutral stance on an item [62] and to maximise reliability and internal validity with additional Likert scale options [63]. Recent research has shown good internal consistency in the general population (Cronbach’s alpha = 0.94) [64]. A higher SCS-R score indicates a higher perceived sense of social connectedness.

### 2.4. Statistical Methods and Software

Participants’ SMU and perceived FoMO, MWB, and SC were measured before and after a seven-day abstinence period. We analysed the data through SPSS, version 26. Alpha values of 0.05 were considered statistically significant.

#### 2.4.1. Pearson’s Correlations

Assumptions were met for correlational analysis. Participant change scores were computed in SPSS to detect significant relationships between total SMU before abstinence (baseline SMU; *M* = 10.5, *SD* = 11.01) and changes in the dependent variables FoMO, MWB, and SC before and after abstinence (change scores). Change scores were calculated for each participant using post-abstinence minus pre-abstinence scores, where higher change scores denoted a higher increase in the dependent variable from pre- to post-abstinence, and negative change scores conveyed a decrease in the dependent variable.

#### 2.4.2. Paired Samples *t*-Tests

To test whether the data met the assumptions of a paired samples *t*-test, histograms and skewness and kurtosis statistics run on SPSS demonstrated approximate normal distribution for each dependent variable in the two related groups (pre-abstinence and post-abstinence), and no significant outliers [65]. During the data cleaning process in Microsoft Excel, scale totals for FoMO, MWB, and SC were computed for each participant at pre- and post-abstinence and these totals were used in data analysis.

#### 2.4.3. Kruskal–Wallis H Tests

Non-parametric tests were performed in SPSS due to data violations of multivariate normality. Participants’ data were re-organised in Microsoft Excel in ascending order by their total SMU throughout the seven-day period before abstinence and from there, participants’ data were divided into quartiles before being exported into SPSS (Level 1: *N* = 15, *M* = 1.67 weekly hours, female = 66.67%, male = 33.33%. Level 2: *N* = 15, *M* = 4.85 weekly hours, female = 80%, male = 20%. Level 3: *N* = 16, *M* = 9.75 weekly hours, female = 62.5%, male = 37.5%. Level 4: *N* = 15, *M* = 25.73 weekly hours, female = 60%, male = 40%). Due to the total of participants being a prime number (*N* = 61), categories were not able to be split into exact quartiles. However, the decision for category 3 to include the extra participant was based upon the intent to try to ensure the female to male ratio was as even as possible in each SMU category. Change scores were used for dependent variables FoMO, MWB, and SC and three separate Kruskal–Wallis H tests were run for each dependent variable. Distributions of FoMO, MWB, and SC change scores were not similar for all groups, as assessed by visual inspection of a boxplot and therefore comparisons of distributions were made as opposed to comparisons of means.

#### 2.4.4. Mann–Whitney U Test

A non-parametric test was performed in SPSS due to data violations of multivariate normality. Gender was categorised into 1 = male (*N* = 20; 32.79%), and 2 = female (*N* = 41; 67.21%). Change scores were used for dependent variables FoMO, MWB, and SC. The two groups of the independent variable, males and females, had differently shaped distributions of the dependent variables as assessed by visual inspection and therefore inferences about differences in distributions were made between the two groups as opposed to differences in medians.

#### 2.4.5. Wilcoxon Signed Ranks Test

A non-parametric test was performed in SPSS due to data violations of normality and symmetry. As tied ranks occurred, continuity correction was applied. For each participant, their mean times spent on their smartphone per day for the seven-day period before and after abstinence were compared.

### 2.5. Thematic Analysis

Thematic analysis was used to analyse the qualitative written data collected in the online questionnaires at both T1 and T2. The data were re-read multiple times for familiarity and preliminary codes were assigned to describe the content. Patterns were then detected to produce themes and these were reviewed multiple times to ensure consistency.

## 3. Results

There was a significant large-sized positive correlation (*r* = 0.550, *p* < 0.01) [66] between SC change scores and MWB change scores: participants’ increase in perceived SC following abstinence was positively related with their increase in perceived MWB following abstinence.

There was a significant medium-sized negative correlation (*r* = −0.340, *p* < 0.01) [66] between FoMO change scores and SC change scores: participants’ decrease in perceived FoMO following abstinence was negatively related with their increase in perceived SC following abstinence.

### 3.1. Abstinence Effects

To compare participants’ perceived FoMO, MWB, and SC before and after SM abstinence, three paired samples *t*-tests were conducted with a Bonferroni-adjusted alpha level of 0.017 (adj. α = 0.05/3). Table 2 shows participants’ mean scores and standard deviations for FoMO, MWB, and SC before and after abstinence. MWB and SC mean values increased after abstaining from SM for seven days in comparison with before abstinence. The mean value for FoMO decreased after abstaining from SM for seven days.

There was a significant decrease in participants’ perceived FoMO scores before and after abstinence; *t*(60) = 4.84, *p* < 0.001, with a medium-sized effect (*d* = 0.55) [67].

There was a significant increase in participants’ perceived MWB scores between before and after abstinence; *t*(60) = −5.19, *p* < 0.001. This difference represented a medium-sized effect (*d* = 0.47) [67].

There was a significant increase in participants’ perceived SC scores before and after abstinence, *t*(60) = −4.13, *p* < 0.001. This difference represented a small-sized effect (*d* = 0.28) [67].

### 3.2. Differences between FoMO, MWB, and SC Change Scores in Level of SMU and Across Genders

Three Kruskal–Wallis H tests were run for each dependent variable to determine any differences in FoMO, MWB, and SC change scores between groups that differed in their level of SMU before abstinence: 1 (lowest), 2, 3, and 4 (highest) SMU level groups. There were no significant differences in participants’ level of SMU before abstinence in FoMO, MWB, and SC change scores.

A Mann–Whitney U test was run to determine whether there were differences in FoMO, MWB, and SC change scores between males and females in the dataset. There were no significant differences in males and females in FoMO, MWB, and SC change scores.

### 3.3. Mean Hours per Day Spent on Smartphone before and after Abstinence

A Wilcoxon Signed Ranks Test with continuity correction was conducted. Table 3 demonstrates the median decrease in participants’ mean hours spent on their smartphone per day before and after abstinence. This was statistically significant, with a median of 3.5 h per day spent on smartphones during seven days of SMU, compared with a median of 2.5 h per day spent on smartphones during seven days of SM abstinence; *z* = −4.885, *p* < 0.001. Using the effect size calculator for non-parametric tests, the effect size was found to be small (*eta*^2^ = 0.39) [68]. Participants spent less time on their smartphones when they were not using SM.

Altogether, the results show that there was an increase in perceived MWB and SC and decrease in perceived FoMO and hours spent on smartphones after SM abstinence. MWB and SC change scores were positively related and FoMO and SC change scores were negatively related.

### 3.4. Thematic Analysis

**Figure 1:**
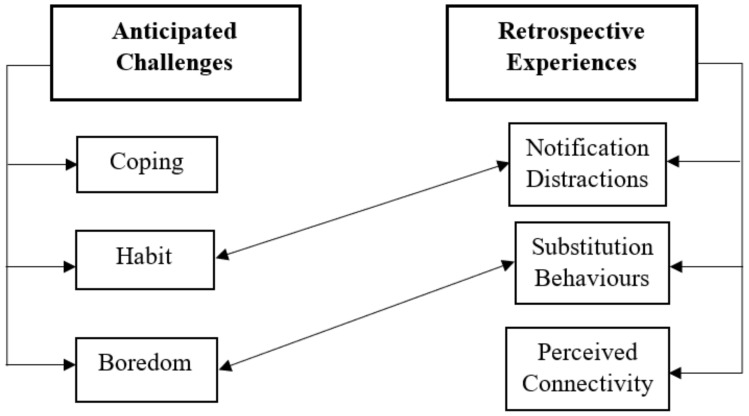
Themes before and after abstinence.

Figure 1 displays the final codes, distinguishing between before and after abstinence codes, where before abstinence codes depict participants’ anticipated challenges of SM abstinence, and after abstinence codes depict participants’ retrospective experiences of SM abstinence.

#### 3.4.1. Coping

This theme captures participants’ main motivations for accessing SM. One of the anticipated challenges of the seven-day abstinence was that SM could not be accessed when wanting to avoid a situation. For example, P36 stated:

*“[Social media abstinence] will challenge me in terms of bringing to light how much I rely on using my phone and scrolling through social media as an escape from situations that I find tricky or simply from having to think about what is happening around me in that moment.”*

SM was used as coping mechanism to escape thoughts and avoid difficult environments. Participants’ concerns about the challenges of the seven-day abstinence centred around being unable to access SM as a way of coping with daily stresses, revealing their reliance on the distractions that SM platforms provide. Further, participants used SM to cope and to find an escape from any difficult situations in their offline world.

#### 3.4.2. Habit

Many participants were concerned that they may fail to fully abstain for seven days due to their SM access being a habitual tendency:

P27: *“I think it will be challenging in the sense that it has become such a habit of mine to passively browse on social media…”*

Participants anticipated that abstaining from SM successfully would be one of the main challenges of the experiment due to instinctive use. The nature of automatically reaching for SM describes participants’ habitual cycle to seek constant stimulation. Further, this participant’s acknowledgement that their habitual behaviour is “passive” suggests the presence of compulsive actions.

#### 3.4.3. Boredom

This theme emerged as another anticipated challenge of the trial. Participants were concerned about being bored as SM was used to fill their time or entertain themselves:

P13: *“…I think social media is a quick and easy (and potentially lazy) way of saving yourself from boredom because there’s content available to you to keep your mind engaged when there’s nothing else around…”*

Here, P13 provided insight as to why they turned to SM in times of boredom, describing it as a quick and easy way for their mind to be engaged with readily available content when there was no other entertainment available at that time.

Participants described boredom as an anticipated concern of abstaining from SM for seven days, revealing how much time they spend on SM as well as their reliance on the platforms in vacant times. This may pose questions regarding how individuals process the world around them if they seem unable to embrace boredom and instead have to constantly strive for entertainment.

#### 3.4.4. Notification Distractions

It seems participants’ anticipations about the challenges of their habitual SM use were correct. This code encapsulates the outcome of the habitual challenge of SM notifications. P34 referred to how turning off notifications helped them to complete the seven days abstinence:

*“…I turned off notifications from these apps and also logged out which I think also helped abstain…”*

This participant also indicated that notifications caused them to experience more FoMO: *“[I] may turn off notifications as they make me feel more like I’m missing out when this is likely to not be true”*.

It appears that participants’ retrospective reflections of the seven-day abstinence trial were that notifications interfered with their abilities to abstain, causing them to automatically open SM, and even instilling a fear of missing out. The experiment caused participants to understand the distracting nature of notifications, with some participants wanting to permanently switch off their notifications. This suggests that the receipt of notifications may trigger habitual SM access due to their distracting and stimulating nature.

#### 3.4.5. Substitution Behaviours

During the trial, participants found that the absence of SM in times of boredom was challenging. Participants found themselves needing to fill this time with other digital applications:

P5: *“…I feel it was more challenging in the beginning as it was the first thing I would do in the morning or something I would do in my free time when I had a moment to spare. I managed to change that behaviour with something else (i.e., games, iBooks, etc.) …”*

P31: *“…It was weird not having social media when I was bored/waiting for things. Usually I’d go on social media in these times just for something to do. So I had to find other things to do instead... Ended up on Google a lot more instead though. Oops!”*

Participants revealed that during boredom and vacant times throughout SM abstinence, they tended to substitute SM with another application, suggesting that other applications may equally satisfy their boredom. Therefore, technology gratification may have less to do with SM-specific platforms and more with having an online entertainment platform.

#### 3.4.6. Perceived Connectivity

Post-abstinence findings show there were mixed feelings on participants’ perceived connectivity. Some participants felt more connected to their offline friends:

P9: *“…I am more interested in updates from friends, which I didn’t see much of anyway before the fast…I know too much of it [social media] can have a negative effect, and you miss too much of what’s going on in the real world around you…”*

Other participants, including P5, felt less connected to their online friends and were concerned of the impact of abstaining from SM:

*“…I feel a bit less connected to my online friends…” “…I am not sure what impact this week has had on our online relationship…”*

Participants’ responses here suggest that perceived connectivity for some is linked with their offline world, whereas for others it is linked with their online friends. This theme appears to consist of differing perspectives of what one’s perceived connectivity is. There appears to be a distinction between “online social connectedness” and “offline social connectedness”.

## 4. Discussion

This study sought to compare participants’ perceived FoMO, MWB, and SC, and smartphone use after seven days of normal SMU (before abstinence) and after seven days of SM abstinence (after abstinence). Results revealed no significant relationship between baseline SMU and FoMO, MWB, and SC change scores. However, a significant positive relationship was found between MWB change scores and SC change scores, and a significant negative relationship was found between FoMO change scores and SC change scores. A significant decrease in FoMO and a significant increase in MWB and SC were revealed after SM abstinence. The results showed that participants’ mean hours per day spent on their smartphone significantly decreased during SM abstinence. Furthermore, thematic analysis results provided insight into the motivations for SMU being coping, habit, and boredom, posing anticipated challenges for participants in the absence of SM. Retrospective experiences of SM abstinence revealed that SM notifications posed a challenge in abstaining due to their distracting nature, causing participants to absent-mindedly access SM as a habitual response. Moreover, it was discovered that to relieve boredom, some participants engaged in substitution behaviours through accessing non-SM applications. Finally, thematic analysis revealed mixed views on perceived connectivity in the absence of SM, with some participants feeling less connected to online peers, and others finding themselves more interested in offline connections during abstinence. Additional analyses revealed no significant differences between different levels of SMU and no significant differences between males and females in FoMO, MWB, and SC change scores.

As a medium-sized effect [67], decreased FoMO following SM abstinence is strongly supported by previous research, suggesting that SMU exacerbates FoMO, specifically with the receipt of SM notifications [4,29]. This is also supported by thematic analysis findings, where participants indicated that the receipt of notifications was distracting, causing them to habitually access SM, and the very receipt of these updates triggered FoMO. Participants in this study also described their habitual SM scrolling as motivation to strive for constant stimulation. In his recent work, Griffiths [69] confirms that as intermittent and unlimited rewards, receiving SM notifications can lead to habitual SMU to avoid missing out on the potential for a psychologically stimulating reward. These results demonstrate the reciprocal process of SMU and FoMO [15,27,30] and can be broken with a period of SM abstinence and disabling notifications. This confirms that SMU drives FoMO more so than FoMO drives SMU [26], but more specifically, SM notification rewards drive FoMO. The saying ‘out of sight, out of mind’ may explain why abstinence reduced FoMO, aided by turning off or muting notifications which assisted in relieving the challenge of abstaining, where reduced cognitive load may have allowed participants to focus on other activities [70]. These findings support literature on the distracting nature of notifications and the negative effects on task performance [71]. Furthermore, the positive experience of removing notifications appears to have had a lasting impact on participants, where many stated they may continue to keep their SM notifications muted, indicating this led to a tangible positive change in their wellbeing.

Increased MWB after abstinence compared with use had a medium-sized effect [67], supported by previous literature suggesting that face-to-face interactions benefit MWB over online interactions [33,34,42] and further supporting research demonstrating associations between SMU and lower MWB [25,50,51]. Research linking SMU to increased social comparisons may explain increased MWB following an abstinence period, where participants are less exposed to information about others’ lives which negatively affect self-contagion [25]. Although offline social comparisons in the offline environment are inevitable as a natural tendency [72], Walther et al.’s [73] model of computer-mediated communication supports components of SM contribute to a sense of identity, serving components such as self-representation. This suggests that SM environments are more likely to elicit negative comparisons than offline environments. Furthermore, thematic analysis findings suggested that SM was used passively and characterised by habitual scrolling, increasing exposure to content where negative social comparisons can be made [25]. Thematic analysis revealed participants used SM to cope, escaping from stressful real-life situations. In the absence of SM during their abstinence period, participants may have been provided with a healthy avenue to process their offline experiences rather than using SM as an easy escape [74].

Although a small-sized effect [67], there was a significant increase in SC as a result of SM abstinence, in line with prior research suggesting that individuals feel more socially connected in the absence of SM [38]. Thematic analysis findings may contribute to building a theory behind why the effect was only small sized as it revealed mixed feelings regarding perceived connectivity, where some participants felt more socially connected offline, while others expressed concerns over the loss of online SC to peers during abstinence, demonstrating individual differences in perceived SC. Perhaps those who experienced lower SC without SM used these platforms to overcompensate for perceived lack of offline connectedness, which may explain the mixed feelings in perceived connectivity as a result of abstinence [75,76]. Although individuals find the connectivity aspect of SM appealing [30,35], prior research suggests that people perceive offline connections as more important than online connections [38]. Moreover, the large-sized [66] positive relationship between SC and MWB change scores is supported by literature indicating the strong relationship between wellbeing and a sense of connectedness [77]. The medium-sized [66] negative relationship between SC and FoMO change scores suggests that participants’ increase in SC was related to their decrease in FoMO following abstinence. Likewise, participants’ decrease in SC was related to their increase in FoMO. This may contribute to the explanation of mixed feelings on perceived connectivity after abstinence, where FoMO could be linked with the online world for some participants, and the offline world for others. The area of perceived connectedness in relation to SMU and SM abstinence warrants further research.

Non-significant differences in participants’ levels of SMU before abstinence and across genders in MWB, FoMO, and SC change scores suggest that significant improvements in MWB and FoMO occurred regardless of whether participants were considered as ‘higher-level’ or ‘lower-level’ SM users and regardless of whether they were male or female. This may be surprising as differences across genders in both substance and behavioural addictive patterns are known [78], with females more likely to develop addictive SM behaviours for social interaction purposes and males more likely to develop compulsive use of more asocial and solitary activities [79,80,81]. This suggests that reasons behind overall improved wellbeing in this study following abstinence may have differed between genders due to the differences in genders in motivations for SMU. Moreover, the results present implications that periods of SM abstinence are beneficial regardless of whether individuals use these platforms in moderation or more frequently. Furthermore, correlational results showed no relationship between baseline SMU before abstinence and changes in FoMO, MWB, and SC. This is contrary to previous literature indicating that individuals who use SM more will experience greater positive outcomes than lower users of SM [24,33,42].

Following abstinence, there was a significant decrease in participants’ mean hours per day spent on their smartphones, as predicted, supporting prior research on the prevalent use of smartphone devices to access SM [3,13,82], where SM abstinence may have instigated less frequent smartphone use, suggesting the influence of smartphone use on desire for constant connectivity [83]. However, this effect was small sized [68]. Substitution behaviours revealed in thematic analysis may provide an explanation, where during abstinence, participants found they substituted their need to fill time with scrolling on other non-SM smartphone applications. Chambers [84] makes the distinction between content gratification and process gratification, where process gratification has less to do with SM content, but the process of accessing SM for entertainment or escapism, suggesting that habitual behaviours derive not from SM content, but the process of scrolling with the finger to occupy the mind [44]. Although participants substituted behaviours, FoMO, MWB, and SC changes were significant, suggesting that habitual SM scrolling behaviours may have a more negative impact on wellbeing than scrolling on non-SM applications, supported in Wilcoxon et al.’s [85] study of smartphone abstinence where participants’ mood and anxiety remained unchanged. This suggests that negative outcomes of SMU may be related to negative exposure to SM content [25,72]. However, Turgeman et al. [86] found that social anxiety and excessive smartphone use were positively correlated, explaining some participants’ needs to substitute SM with other smartphone applications. Furthermore, the positive changes in FoMO, MWB, and SC may have been moderated by the decrease in smartphone use during SM abstinence, warranting further research.

However, these positive changes may be explained by social cognitive theory of mass communication, suggesting that people’s views of SM are influenced by news reports, which mostly contain negativity surrounding SM [87]. Further, the ‘digital cleanse’ is becoming a more popular phenomenon particularly amongst celebrities taking abstinence breaks from technology [20] and self-optimisation theorises that participants may have felt morally better in successfully abstaining. Nonetheless, thematic analysis revealed that the voluntary sample still found abstaining challenging, regardless of underlying motivations for taking part in the experiment, demonstrating strong evidence that SM abstinence can induce positive changes in psychosocial behaviours compared with SMU. Furthermore, positive significant changes in FoMO, MWB, and SC were not related to initial degree of SMU, adding more weight to the results.

In a smartphone SM pervasive society [1,2], this study has important implications for demonstrating the importance of unplugging from SM at times, supporting the benefit and feasibility of an abstinence period of seven days [43]. With little known about social media abstinence [38], the present mixed-methods research draws compelling comparisons between the effects of SMU and SM abstinence on psychosocial behaviours. Moreover, using digital applications to measure multiple platform SMU increased the accuracy of results and the thematic analysis offered theoretical explanations for behavioural changes elicited by the abstinence period. The present results have wider implications for individuals and society in supporting the benefits of a short SM abstinence period as an intervention to manage the potential negative effects of SM and improve wellbeing [25,30]. The results also offer useful suggestions for parents in managing their children’s screen time, further contributing to educational and health research [88].

Some limitations are worth noting. First, the participants’ age range between 20 and 49 years suggests that both students and individuals in employment have been included, and this may have an important influence on both SMU and coping with abstinence where younger age groups are more likely to use SM more frequently and for social interaction purposes as well as self-disclosure, and acquire a larger network than older age groups. However, the sample mostly consisted of a younger population (*M* age = 24.44); an abstinence period for an older age group may yield different results due to the different uses of SM across generations [82]. Second, the findings present a further question of whether the significant reduction in smartphone use during abstinence moderated the positive changes in FoMO and MWB. Finally, the thematic analysis findings of participants’ mixed feelings on perceived connectivity during abstinence coupled with an increase in SC after abstinence warrants further exploration as to whether there were other differences between those who felt more socially connected after abstinence and those who felt less connected. Future research should focus on investigating motivations for SMU across generations and genders, and explore individual differences in perceived online social connectivity and offline social connectivity. The extent to which reduction in smartphone use during SM abstinence moderates positive changes in wellbeing needs exploring, and psychosocial behavioural effects should be measured across a longer abstinence period, which may be useful in determining whether results are lasting or are due to initial positivity derived from self-optimisation.

## 5. Conclusions

This study’s findings demonstrate that a seven-day SM abstinence period significantly increased perceived MWB and SC, and significantly decreased perceived FoMO in comparison to a seven-day period of SMU. However, SM abstinence elicited only a small-sized increase in perceived SC, with some participants experiencing higher perceived SC through the online world and others experienced higher perceived SC through the offline world. Furthermore, there were no differences between males and females and higher- and lower-level SMU on FoMO, MWB, and SC change scores, suggesting that the overall improvement in wellbeing after abstinence occurred regardless of gender and SMU level. This research also provides insight into the motivations for SMU, suggesting that the process of scrolling as a time filler is a key motivator. The findings also support research on the distracting nature of notifications, suggesting that SM notifications trigger FoMO. Findings further provide evidence of the importance of unplugging at least for temporary periods and are useful for education and health research in promoting balanced SMU and SM abstinence.

## Figures and Tables

**Table 1:** Social media weekly use hours before abstinence across smartphones and other digital devices (i.e., desktop, tablet, and television; *N* = 61).

Social Media Platform	Smartphone	Other	Total
Facebook	148.75	88.75	237.5
YouTube	84	93	177
Instagram	127.25	6.25	133.5
Snapchat	23.5	4	27.5
Reddit	15	12	27
Twitter	19.75	2.75	22.5
Pinterest	3.25	4.5	7.75
Tumblr	2	5	7

**Table 2:** Means and standard deviations for each dependent variable before abstinence and after abstinence, including their change score and Cronbach’s alpha values.

	Mean ± SD		Mean Change ± SD	Cronbach’s Alpha	
	Pre	Post		T1	T2
Fear of Missing Out	24.9 ± 6.25	21.6 ± 5.48	−3.2 ± 5.22	0.78	0.77
Mental Wellbeing	46.1 ± 8.58	50.1 ± 8.18	4 ± 5.97	0.90	0.92
Social Connectedness	81.3 ± 8.2	80.9 ± 6.57	−0.1 ± 6.26	0.93	0.95

**Table 3:** Median and ranks for participants’ change in mean daily smartphone hours before and after abstinence.

	Median		Ranks		
	Pre	Post		*N*	Mean
Mean Time on Smartphone per Day	3.5	2.5	Negative	49	32.2
			Positive	11	23.
			Ties	1	
